# Body Measurement and Health Risk Education Effects on Older Adults’ Health Beliefs and Behaviors

**DOI:** 10.1093/geroni/igaa057.895

**Published:** 2020-12-16

**Authors:** Beatrice Gaynor

**Affiliations:** University of Delaware School of Nursing, Newark, Delaware, United States

## Abstract

Waist circumference (WC) measurement is an indicator of central obesity related disease risk that is rarely used in primary care (PC). The current PC practice of body mass index (BMI) calculation to screen for disease risk lacks specificity to the older adult habitus. Guided by the Health Belief Model, this study utilized a one-way analysis of covariance to examine the effect of experimental cues, WC measurement and central obesity disease risk education, compared to control cues, BMI and obesity classification, on older adults’ health beliefs (perceived susceptibility and health benefits) and behaviors (diet and exercise) 6 weeks post cues/intervention. Of the 99 participants (control group [N=49]; experimental group [N=50]) 92% reported ‘never’ having WC measurement and 76% reported ‘never’ having BMI calculation in PC. Both groups reported high levels of perceived susceptibility and exercise at baseline. Changes in perceived susceptibility, diet, and exercise were non-significant in either group. There was a significant increase in perceived health benefits of WC measurement (p=0.01) and BMI calculation (p=0.01) in the experimental group compared to the control group. Willingness to exercise (p=0.007) significantly increased in the experimental group compared to the control group. The lack of BMI experience in both groups may have caused control cues to function as experimental cues in both groups. Thus, this study provides evidence that combined use of WC measurement, central obesity health risk education, BMI calculation, and obesity classification increase perceived benefits of body measurements and motivate physical activity in older adults over BMI and obesity classification alone.

